# Hand-to-surface bacterial transfer and healthcare-associated infections prevention: a pilot study on skin microbiome in a molecular biology laboratory

**DOI:** 10.3389/fmed.2025.1546298

**Published:** 2025-03-21

**Authors:** Arianna Delicati, Beatrice Marcante, Dolores Catelan, Annibale Biggeri, Luciana Caenazzo, Pamela Tozzo

**Affiliations:** ^1^Legal Medicine Unit, Department of Cardiac, Thoracic, Vascular Sciences and Public Health, University of Padova, Padova, Italy; ^2^Department of Pharmaceutical and Pharmacological Sciences, University of Padova, Padova, Italy; ^3^Unit of Biostatistics, Epidemiology and Public Health, Department of Cardiac, Thoracic, Vascular Sciences and Public Health, University of Padova, Padova, Italy

**Keywords:** human microbiome, forensic genetics, healthcare-associated infections (HAI), public health, bacterial transfer

## Abstract

**Background:**

Healthcare-associated infections (HAIs) are a major global public health problem, contributing significantly to patient morbidity and mortality. This study analyses differences in type and amounts of bacteria transferred from volunteers’ dominant palm to two healthcare-relevant surfaces (glass and laminate table), both before and after hand washing with water and antibacterial soap. The aim was to understand hand-to-surface microbial contamination and support the development of HAI prevention strategies.

**Methods:**

Microbial DNA was extracted and sequenced to identify bacteria species. Taxonomic and statistical analyses were performed to evaluate bacterial diversity and abundance across the experimental groups.

**Results:**

The results confirmed greater bacteria abundance and species richness on palm compared to surfaces, with a significant reduction after hand washing, especially on glass. Taxa analysis highlighted the increased persistence of Gram-negative HAIs-related bacteria on laminate surface, while Gram-positive opportunistic bacteria were more abundant on palms and glass surface. Beta diversity confirmed significant differences in microbial composition between the groups, highlighting the importance of bacteria-surface characteristics in designing preventive measures.

**Conclusion:**

Despite some limitations, our study emphasizes the importance of microbiological surveillance for all opportunistic bacteria with pathogenic potential. These findings can contribute to more effective guidelines for surface disinfection and hand washing, key elements in preventing HAIs.

## 1 Introduction

The skin is the largest organ in the human body, which has the main role of protecting the body against the external environment and potential pathogens (1). The human body hosts trillions of microorganisms, some of which are hosted on the skin forming an ecosystem called “skin microbiota” (1–4). The skin microbiota is shaped by different factors such as diet, sex, habits, disease state, interactions between individuals, and environment (5–11). In particular, there is a bidirectional interaction between the skin microbiota and the environment, with microorganisms being exchanged upon contact (12). For this reason, despite the skin microbiota being relatively stable over time and individual-specific, it represents an interesting field of scientific research and its study may have important consequences in the clinical practice (1, 4, 6, 12–18).

In healthcare environments, where the risk of healthcare-associated infections (HAIs) is particularly high, understanding the dynamics of the skin microbiota and microbial contamination surfaces is very important to prevent the spread of infections and to promote the safety of patients and healthcare workers. In this context, the skin can also act as a reservoir for pathogenic microorganisms, which can be transferred to environmental surfaces during direct or indirect contact (19–22). The transfer by direct contact is defined as the passage of microorganisms from a person directly onto a surface, whereas the transfer by indirect contact involves one or more intermediate steps before reaching the final surface (e.g., one person could transfer its microorganisms to another one if both persons entered in contact with the same object in two different consecutive moments) (23–25).

Understanding the dynamics of microbial transfer, within the healthcare environment, is essential for designing effective prevention strategies for HAIs. In hospital surface microbial contamination, in fact, has been associated with the spread of HAIs, which represents a significant issue for patient safety and infection control in hospital and other healthcare settings (26–30). HAIs, also known as nosocomial infections, are infections that patients contract during their stay in hospitals or in other healthcare facilities. These infections should not be present or incubating at the time of patient admission and typically develop 48 h or more after admission (26, 29–34). The risk of HAIs increases with: invasive procedures such as surgeries or the use of medical devices such as mechanical ventilators, exposure to contaminated environments, inadequately sterilize surfaces or medical equipment, contact with healthcare workers or visitors, and the presence of antibiotic-resistant microorganisms (21, 35–38).

HAIs are a leading cause of illness and death worldwide, particularly in hospitalized and immunocompromised patients. They are often caused by a variety of pathogenic microorganisms known as ESKAPE bacteria, an acronym that includes *Enterococcus* spp., *Staphylococcus aureus*, *Klebsiella pneumoniae*, *Acinetobacter baumannii*, *Pseudomonas aeruginosa*, and *Enterobacter* spp. The ESKAPE bacteria are so named because they are known for their ability to ‘escape’ antibiotics and other conventional treatments, making them extremely difficult to eliminate and causing serious complications in hospitalized patients. These pathogens, together with many others such as *Clostridium difficile*, *Haempophilus* spp., *Rotia* spp., *Stenothrophomonas* spp., and *Streptococcus* spp. are recognized as the main cause of HAIs (29–32, 39, 40).

The effective management of HAIs requires a multidisciplinary approach that includes not only the treatment of the infections themselves, but also the prevention of their spread within healthcare facilities. Therefore, due to the significant clinical and economic impact associated with their treatment and management, numerous studies have focused on the development of effective protocols for the prevention and control of these infections, in order to ensure patient safety. Among the key strategies adopted, there are the rigorous implementation of hand hygiene procedures, the sterilization of healthcare surfaces and medical equipment, the rational use of antibiotics to prevent the emergence of multi-resistant bacteria, the implementation of public health surveillance protocols, the continuous training of healthcare-workers, and the screening and the categorization, as well as the isolation of infected patients (31, 32, 41–45).

Despite the implementation of the previously cited preventative measures, HAIs continue to be a significant public health challenge. Therefore, ongoing efforts are needed to improve infection control practices and develop new strategies to reduce the spread of pathogens and promote the safety of patient and healthcare worker, understanding the dynamics of the skin microbiota and microbial contamination of surfaces. To delve deeper into this intricate network of microbial transfer, there is a need for comprehensive studies exploring the direct transfer of human microbiota into various surfaces. Investigating the microbial load and the specific types of microorganisms present on these surfaces can provide valuable insights into potential transmission routes and contamination hotspots within healthcare settings (21, 46–48). Such knowledge not only aids in the development of targeted cleaning and disinfection protocols but also lays the groundwork for future research and interventions aimed at reducing the risk of HAIs (36, 47).

Therefore, this study has the purpose to contribute to the growing understanding of microbiological transfer processes and bacteria persistence on different surfaces, particularly relevant in prevention of HAIs. Through the analysis of the direct transfer of microorganisms from the volunteers’ dominant hand to these surfaces, both before and after hand washing, we tried to study in detail the adhesion of microorganisms on surfaces and to evaluate the effectiveness of hand washing in reducing their presence. By analyzing the variations in the microbiota deposited on surfaces, a better understanding of the mechanisms of microbial transmission will be gained. The results of this study can contribute to develop strategies aimed at preventing HAIs, thus improving the safety and quality of healthcare system through targeted interventions on the reduction of environmental contamination.

## 2 Materials and methods

### 2.1 Sample collection

The study investigated direct microbial transfer from the dominant hand of different volunteers to two types of surfaces. Transfer was assessed both before and after hand washing with water and antibacterial soap provided by the healthcare facility hosting the forensic genetics laboratory. A total of 19 healthcare-worker volunteers of both sexes (11 females and 8 males) aged between 25 and 64 years were enrolled. The inclusion criteria were being in good general health condition and not having taken antibiotics in the 2 weeks prior to the sampling. Once selected, the volunteers willingly provided written informed consent to participate in the study. Volunteers were asked to maintain their normal daily routine in terms of diet, personal hygiene, and exercise.

To ensure the representativeness of surfaces commonly found in healthcare settings, we chose a glass surface and a laminate table. Both surfaces are non-porous, with the latter designed to further reduce possible microbial growth (49). Before starting with the study, to eliminate any microbial trace from the surfaces, they were first cleaned with a bleach solution diluted with distilled water in a 1:3 ratio. The excess bleach was then removed by rinsing the surfaces with distilled water, following by a final cleaning with 70% ethanol.

The experiment took place in a controlled, closed environment at room temperature. The experiment took place between March 5 and 7, 2024. To further limit confounding variables, we selected three consecutive days with similar weather conditions for sampling the microbial profile of the volunteers under different experimental settings. All the sampling procedures were performed by sliding a sterile swab moistened with sterile physiological water. The experimental procedure of samples collection was therefore performed in three different days at the end of which five experimental groups were produced: volunteers’ dominant palm microbiota (group 1: Palm), microbiota deposit on glass before hand washing (group 2: GlassBHW), microbiota deposit on glass after hand washing (group 3: GlassAHW), microbiota deposit on laminate table before hand washing (group 4: TableBHW), and microbiota deposit on laminate table after hand washing (group 5: TableAHW). In particular, on the first day, microbiota samples were collected from each volunteer by swabbing their dominant palm to establish an individualized reference microbial profile. On the subsequent 2 days, our focus shifted to surface sampling. Day two involved the collection of biological samples from the glass surface. Each volunteer placed their dominant hand on the cleaned glass surface twice, once before hand washing and the other after hand washing. On day three, the activities of day two were repeated, focusing on the laminate table surface. Therefore, at the end of the third day, from each volunteer a total of five samples were obtained, one for each experimental group: one from the palm, two from the microbial samples deposited, respectively, on glass and laminate table before hand washing, and two from the same surfaces but after hand washing.

**Figure 1:**
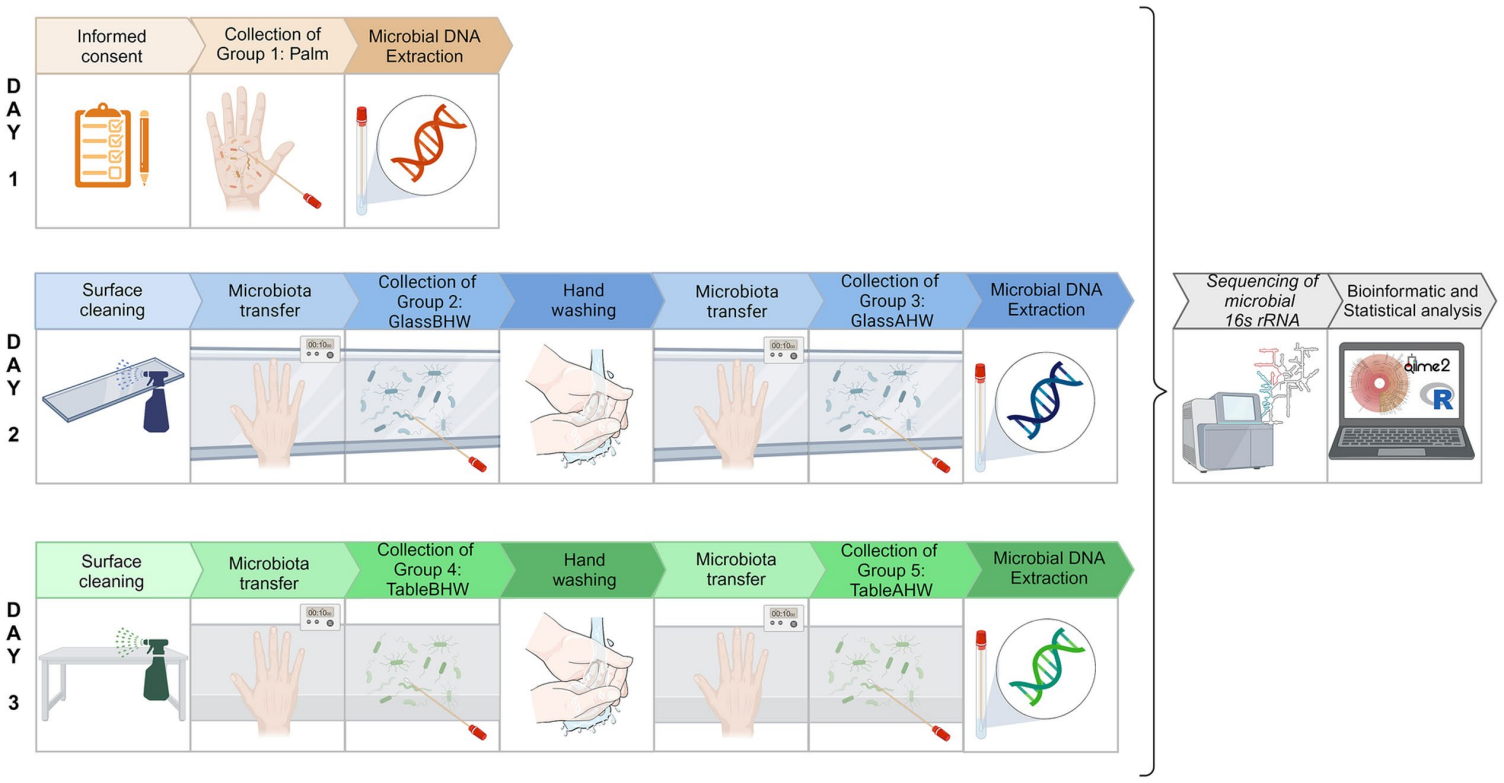
Experimental protocol overview. Schematic representation of the experimental protocol to analyze the skin microbiota of volunteers’ dominant palms and the hand-to-surface microbial transfer, both before and after hand washing. Day 1 represents the collection of palm microbiota (group 1: Palm). Day 2 represents the collection of samples on glass surface: before hand washing (group 2: glassBHW) and after hand washing (group 3: GlassAHW). Day 3 represents the collection of samples on laminate table surface: before hand washing (group 4: TableBHW) and after hand washing (group 5: TableAHW). In group 2, 3, 4, and 5 contact time between the hand placement and the surfaces was of 10 s and the sample collection occurred 3 min after surface microbiota deposition. After microbial DNA extraction, all the samples continued with the downstream analysis simultaneously [Created in BioRender, https://BioRender.com/i54f239].

We specify that, for our experimental setting on day two and three, we decided to maintain a consistent contact time of 10 s between the hand placement and the surfaces, for each condition (50). Samples from surfaces were collected for each volunteer and experimental setting 3 mins after microorganisms were deposited. Once all the samples for each experimental setting were collected, we proceeded with the microbial DNA extraction followed by a detailed analysis to determine the skin microbial profile of the 19 volunteers’ dominant palms and the microorganisms deposited on the surfaces under the different experimental settings. In particular, the microbial DNA extractions, from the samples of each experimental group, were performed together after the collection for that specific group was completed. This process was repeated for each experimental group over 3 days. After each extraction, the microbial DNA samples were stored at −20°C until all extractions were completed. Once all sample collections and DNA extractions were completed, all the extracted microbial DNA samples of all the experimental groups proceeded simultaneously with both laboratory analysis and bioinformatics and statistical analysis (Figure 1).

### 2.2 Microbial DNA extraction and analysis

Microbial DNA was extracted using the QIAamp PowerFecal Pro DNA Kits (QIAGEN, Hilden, Germany) according to the manufacturer’s instructions with a minor modification consisting of the addition of 800 μL of CD1 solution to each swab followed by vortexing for 5 s and centrifugation at 2000 g for 10 min. DNA was eluted in 35 μL of C6 solution and quantified using NanoDrop One Microvolume UV–Vis Spectrophotometer (ThermoFisher Scientific, Waltham, United States).

The extracted microbial DNA was sent to an external facility (Personal Genomics SRL, Verona, Italy) for library preparation, DNA sequencing and an initial bioinformatics analysis. Library preparation was done using primer combination Pro341F and Pro805R to amplify the hypervariable regions V3-V4 of the 16S rRNA. Subsequently, all samples were sequenced with 300 paired-end with an Illumina MiSeq platform (51).

### 2.3 Bioinformatic and statistical analysis

The quality control of raw sequencing reads was performed using FastQC v0.11.9, whereas filtering and denoising was performed using DADA2 (Qiime2 release 2023.9) (52, 53). The reads were quality trimmed at a length of 270 and 245 nt, respectively for read1 and read2. Taxonomy analysis was performed with “qiime feature-classifier classify-sklearn” using the greengenes 2 database (gg_2022_10) (53–55). Further bioinformatics and statistical analysis were performed with Qiime2 (release 2023.9) and R studio (version 4.3.22023.10.31 ucrt) (53, 56, 57). Krona charts to represent the mean relative abundance of the different microorganisms in the different experimental groups (Palm, GlassBHW, GlassAHW, TableBHW, and TableAHW) were produced with KronaTools (58). Stacked bar-graph were produced with Qiime2 (release 2023.9) to graphically represent the relative frequency of all phyla belonging to each sample in the various experimental settings.

The statistical analysis considered within subject comparisons, removing inter-individual variabilities, such as differences in palm size. Therefore, all abundance comparisons to evaluate differences at the different taxonomic levels were performed with the Friedman test given the non-parametric nature of the data (differences were considered significant in case of *p* < 0.05). For post-hoc multiple comparisons of all the experimental groups for each microorganism at the different taxonomic levels (domain, phylum, genus, and species), the Wilcoxon test for paired data with Bonferroni adjustment was performed. The differences were considered significant with an adjusted *p*-value (*p*-adj value = *p*-value*10) < 0.05—Bonferroni adjustment was performed for the number of comparisons made (*n* = 10). Significant results were highlighted in the box plots with jittered points below with asterisks (*) indicating the level of significance: *means *p*-adj value <0.05, **means *p*-adj value <0.01, ***means *p*-adj value <0.001, and ****means *p*-adj value <0.0001.

The mean abundance of bacterial genera associated with HAIs or identified as opportunistic pathogens in the various experimental groups was also showed as bar graph whereas the same pathogenic bacteria, but at species level, which demonstrated statistical significance in multiple comparisons among experimental groups, were visualized using a heatmap to graphically represent the variations in abundance across the samples in the different experimental groups. Eventually, to evaluate the microbial diversity present in different experimental conditions, we used Qiime2 (release 2023.9) (53). We performed an alpha diversity analysis using both the Shannon index and species richness. The Shannon index allows us to quantify the diversity within each sample, considering both the number of species (richness) and the distribution of species abundances (equity), whereas species richness provides a straightforward count of the different species present, focusing solely on the number of species without accounting for their relative abundances. We generated alpha-rarefaction curves for each experimental group, which allowed us to visualize how diversity changes within each experimental setting. Next, for a detailed and comparative assessment of microbial diversity, we created firstly a violine-plot of the Shannon Entropy for various groups of samples and, lastly, we performed a beta diversity analysis through PCoA (Principal Coordinates Analysis) at the species level. This analysis provides a three-dimensional visual representation of the relationships between samples. We generated the beta diversity graphs using Aitchison distance.

## 3 Results

The bacterial persistence on various surfaces and the impact of hand washing were evaluated considering five experimental groups: dominant palm microbiota from volunteers (group 1: Palm), microbiota deposit on glass before hand washing (group 2: GlassBHW) and after hand washing (group 3: GlassAHW), and microbiota deposit on laminate table before hand washing (group 4: TableBHW), and after hand washing (group 5: TableAHW).

The first analysis was conducted with the aim of visually evaluating the differences in the mean relative abundances between the experimental groups at all taxonomic levels. To achieve this purpose, Krona charts were produced for each experimental group (Supplementary Figures 1a–e) (58). Krona charts organize relative abundances in sectors with a very precise hierarchy, which goes from inside to outside according to decreasing taxonomic levels: from d-Domain, p-Phylum, c-Class, o-Order up to f-Family, g-Genus to s-Species.

**Figure 2:**
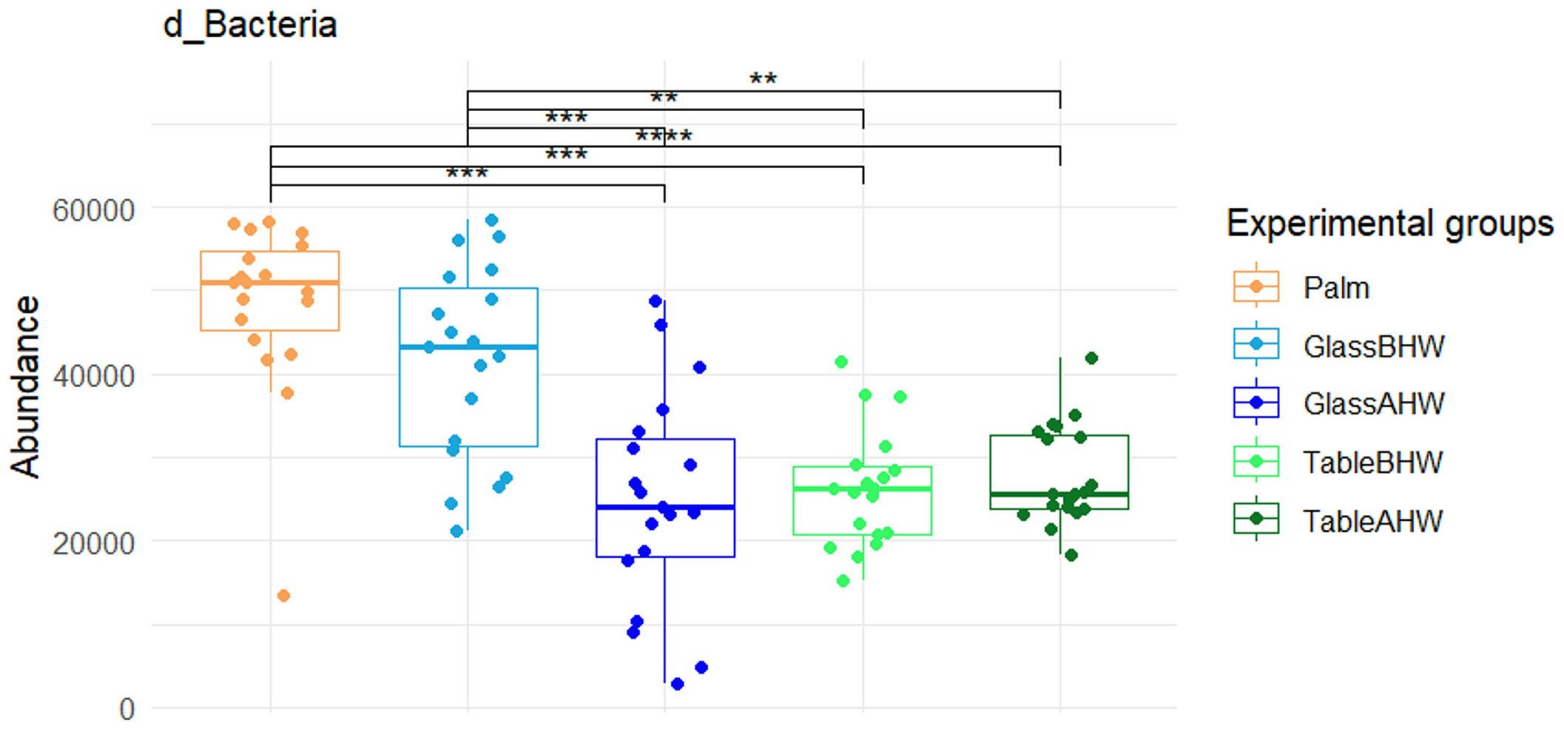
Comparison of the abundance of the domain Bacteria across various experimental groups. This graph shows the comparison between the amount of Bacteria among all the different experimental groups. If significant, the values are indicated as follows: *p-adj value <0.05, **p-adj value <0.01, ***p-adj value <0.001, and ****p-adj value <0.0001.

From the Krona charts (Supplementary Figures 1a–e) it is possible to observe, as expected, that practically 100% of the DNA sampled from the 19 volunteer subjects is of bacterial origin. Comparing the abundance of Bacteria across experimental groups with the Friedman test a significant difference was found (*p* < 0.0001). In particular, after post-hoc comparisons by Wilcoxon test for paired data with Bonferroni adjustment, the bacterial counts appeared to be significantly higher on the palm of the hand compared to all the other experimental groups and, in the case of glass, a significant reduction was observed after hand washing compared to the case of the laminate table, which showed lower level of abundance even before hand washing (Figure 2).

To obtain deeper insight, we analyzed mean relative abundance at each taxonomic levels, primarily focusing on phyla, genera, and species. Here we reported the results relating to taxonomic elements with relatively high abundances and/or significant differences among experimental groups (full results available on request). Therefore, comparison among experimental groups were not shown for bacteria with a mean abundance below 200 units and/or without significant differences among the experimental groups (Friedman test and Wilcoxon test for paired data with Bonferroni adjustment).

The relative bacterial percentages at the phylum level are represented in the Krona charts (Supplementary Figures 1a–e). Qualitatively, the graph shows a clear prevalence of *Proteobacteria* in the TableBHW and TableAHW groups, and of *Actinobacteriota* and *Firmicutes D* in the other groups. In particular, *Proteobacteria* have an average relative frequency of 22, 28, 34, 81, and 85% in Palm, GlassBHW, GlassAHW, TableBHW, and TableAHW, respectively. *Actinobacteriota* exhibit an average relative frequency of 40% in Palm, 31% in GlassBHW, 30% in GlassAHW, 6% in TableBHW, and 3% in TableAHW. Meanwhile, *Firmicutes D* display an average relative frequency of 25% in Palm, 27% in GlassBHW, 17% in GlassAHW, 3% in TableBHW, and 3% in TableAHW.

**Figure 3:**
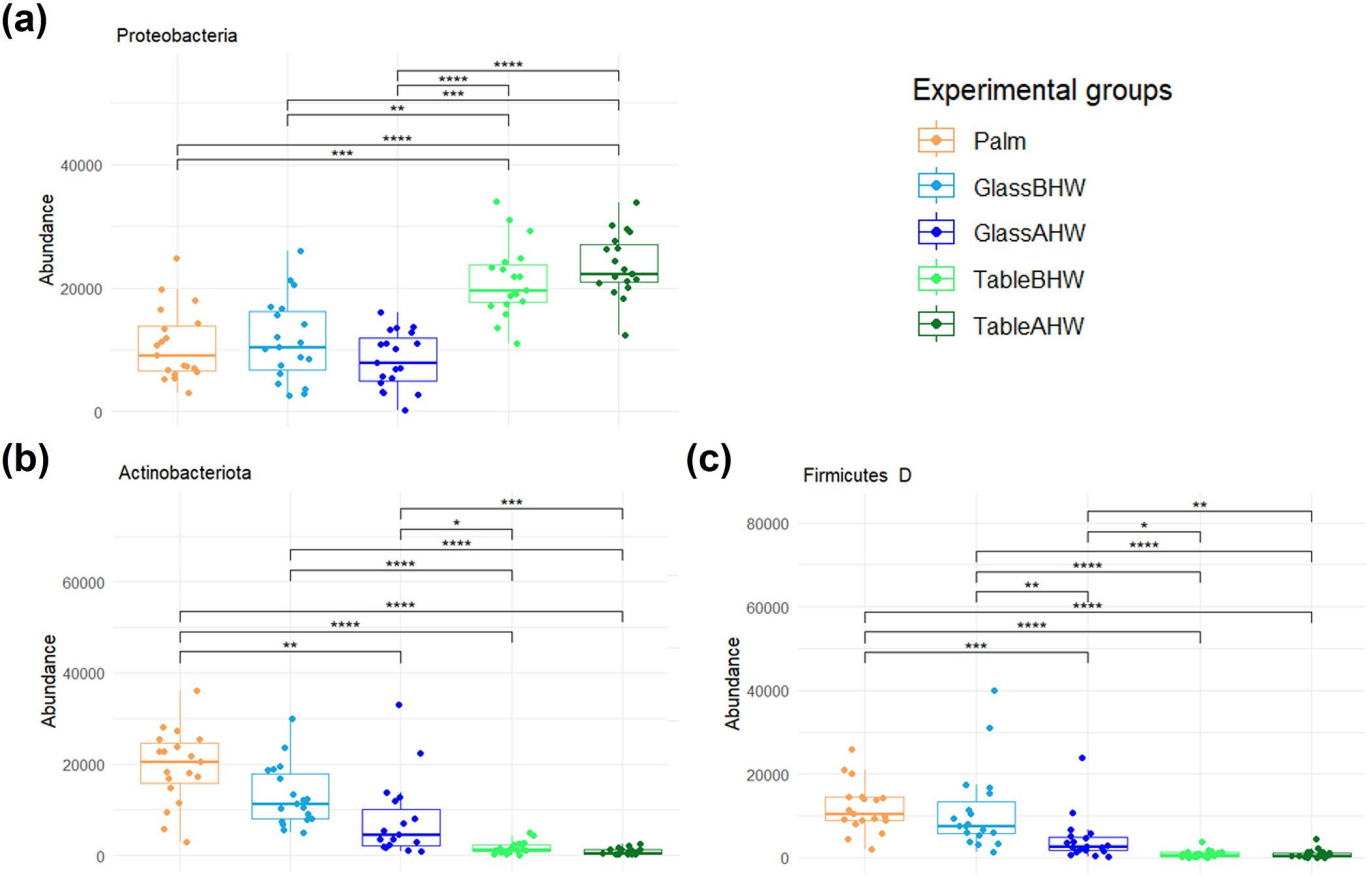
Comparison of the amount of the most abundant phyla among experimental groups. These graphs illustrate the differences in the abundance of the **(a)** *Proteobacteria*, **(b)** *Actinobacteriota*, and **(c)** *Firmicutes D* across various experimental groups. If significant, the values are indicated as follows: *p-adj value <0.05, **p-adj value <0.01, ***p-adj value <0.001, and ****p-adj value <0.0001.

The differences in abundance of these three main phyla by experimental groups were analyzed by the Friedman test followed by Wilcoxon test for paired data with Bonferroni adjustment. Statistically significant differences in abundance were observed in relation to the surface from which the bacteria were collected. A greater similarity was confirmed between palm and glass, particularly when considering the GlassBHW (Figures 3a–c).

**Figure 4:**
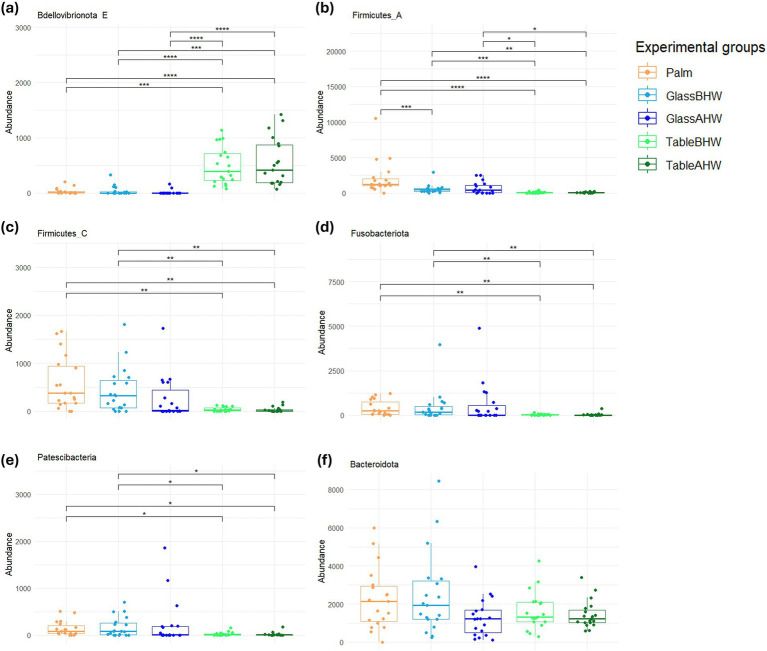
Comparison of the amount of the low abundant phyla among experimental groups. These graphs illustrate the differences in the abundance of the **(a)** *Bdellovibrionota E*, **(b)** *Firmicutes A*, **(c)** *Firmicutes C*, **(d)** *Fusobacteriota*, **(e)** *Patescibacteria*, and **(f)** *Bacteroidota* across various experimental groups. If significant, the values are indicated as follows: *p-adj value <0.05, **p-adj value <0.01, ***p-adj value <0.001, and ****p-adj value <0.0001.

The relevant results of the analyses of all the remaining phyla were reported on Figures 4a–f. In particular, with the only exception of the phylum *Bdellovibrionota E* (Figure 4a), the phyla show a similar tendency (Figures 4b–f). In particular, in *Firmicutes A*, *Firmicutes C*, *Fusobacteriota*, and *Patescibacteria* (Figures 4b–e) the abundance of the bacterial samples collected from the palms is very similar to those collected in the case of GlassBHW and GlassAHW; in the GlassAHW group there is a slight decrease in abundance; and a more significant reduction in the amount of these phyla was observed in the samples collected from laminate table, both before and after hand washing. Instead, the *Bacteroidota* phylum, despite the similar tendency to the other phyla, is highly represented in the different experimental groups, without significant differences between them (Figure 4f). Considering the phylum *Bdellovibrionota E*, a greater abundance was observed in the samples taken from the laminate table before and after hand washing, compared to the other groups (Figure 4a).

**Figure 5:**
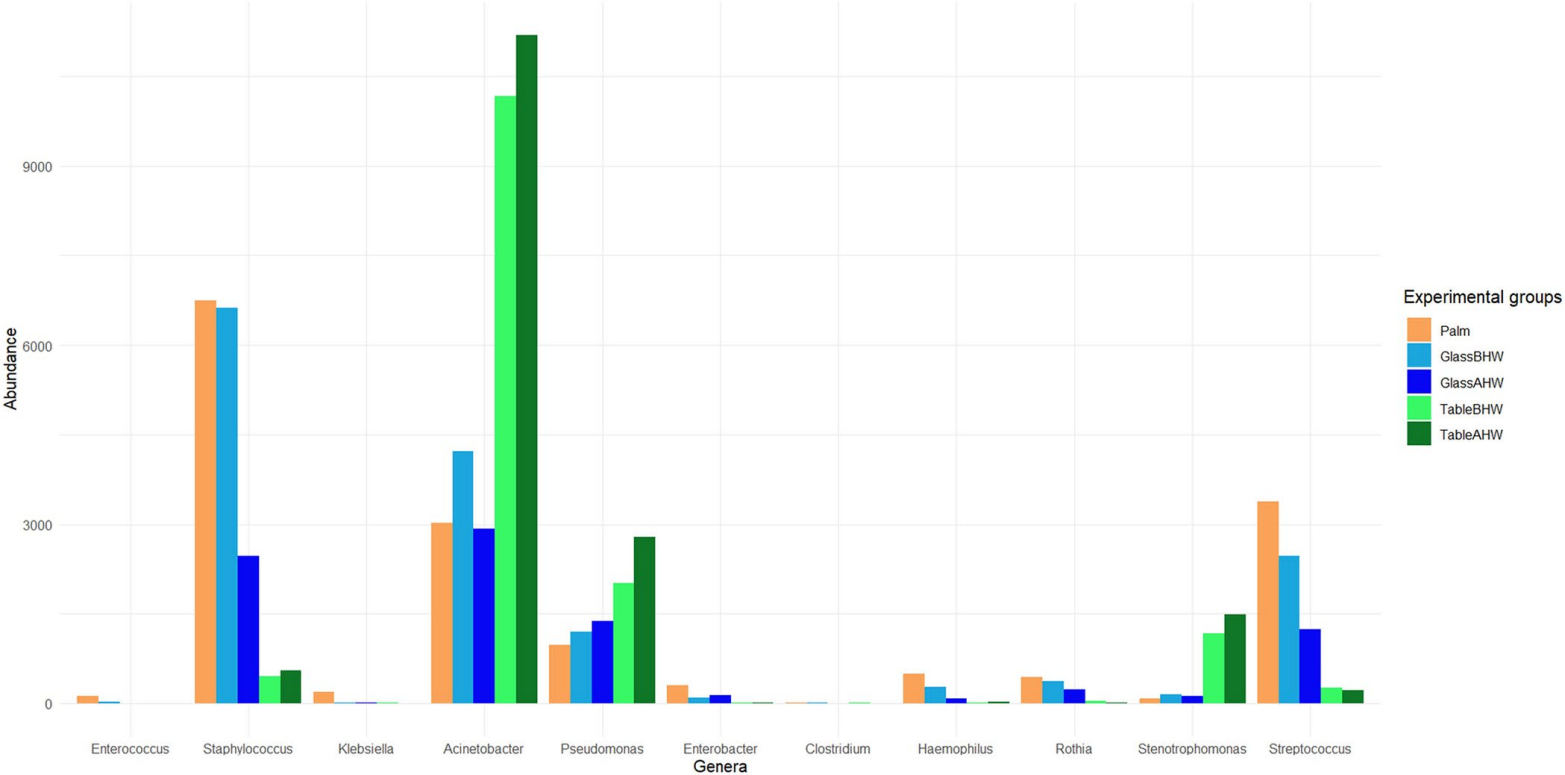
Bacteria at genus level associated with HAIs. The graph shows HAIs-related bacteria and their average abundance across the experimental groups.

At the genera level the results were reported considering the bacterial genera associated with HAIs and in particular those reported in the HAIs prevalence document produced by the European Center for Disease Prevention and Control relatively to the years 2022 and 2023 (26, 30). Figure 5 shows the average abundance of HAIs-related genera detected in our samples. The amount of Enterococcus, *Klebsiella*, and *Clostridium*, such as those of others HAIs-related bacterial genera, is so low that these genera cannot be considered relevant in this study. Other genera which always resulted of low abundance are *Enterobacter*, *Haemophilus*, and *Rothia*, however these genera showed a slightly higher persistence in the samples of Palm, GlassBHW, and GlassAHW. On the contrary, *Staphylococcus*, *Acinetobacter*, *Pseudomonas*, *Stenotrophomonas*, and *Streptococcus*, were detected in high amount on our samples, exhibiting consistent differences in adhesion propensities depending on the considered surface (Figure 5).

**Figure 6:**
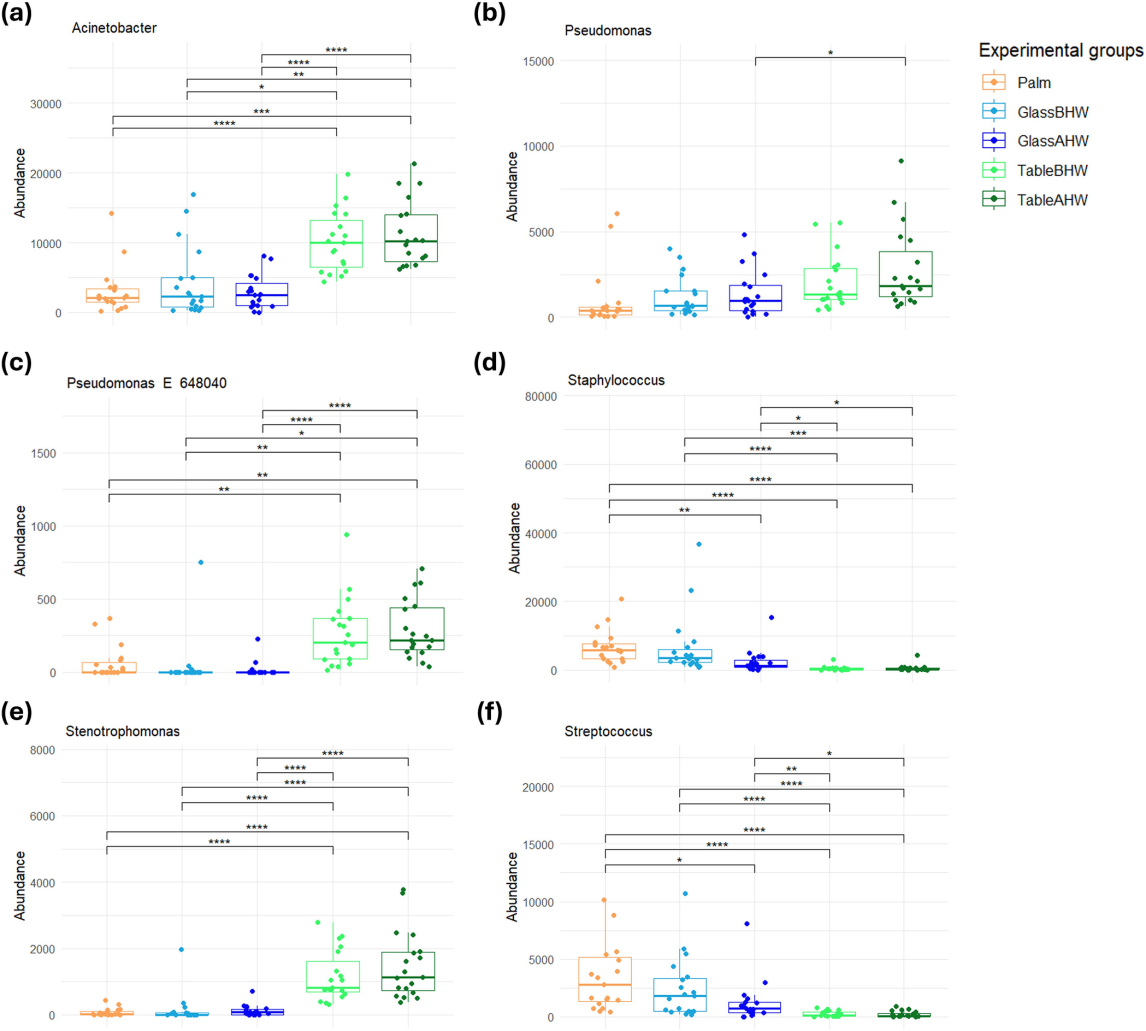
Comparison of the amount of the most abundant genera related to HAIs among experimental groups. These graphs illustrate the differences in the abundance of the **(a)** *Acinetobacter*, **(b)** *Pseudomonas*, **(c)** *Pseudomonas E 648040*, **(d)** *Staphylococcus*, **(e)** *Stenotrophomonas*, and **(f)** *Streptococcus* across various experimental groups. If significant, the values are indicated as follows: *p-adj value <0.05, **p-adj value <0.01, ***p-adj value <0.001, and ****p-adj value <0.0001.

In particular, *Staphylococcus* and *Streptococcus* are predominant on glass both before and after the hand washing, whereas *Acinetobacter*, *Pseudomonas*, and *Stenotrophomonas* were predominant on the laminate table in both conditions. The statistical analysis highlighted significant differences among experimental groups (Figures 6a–f), mostly in the genera of *Acinetobacter*, *Staphylococcus*, *Stenotrophomonas*, and *Streptococcus* (Figures 6a,d,e,f). Regarding the genus *Pseudomonas*, no large overall differences were observed, but a specific clade, identified as *Pseudomonas E 648040*, showed greater deposition on the laminate table, both before and after hand washing (Figures 6b,c).

**Figure 7:**
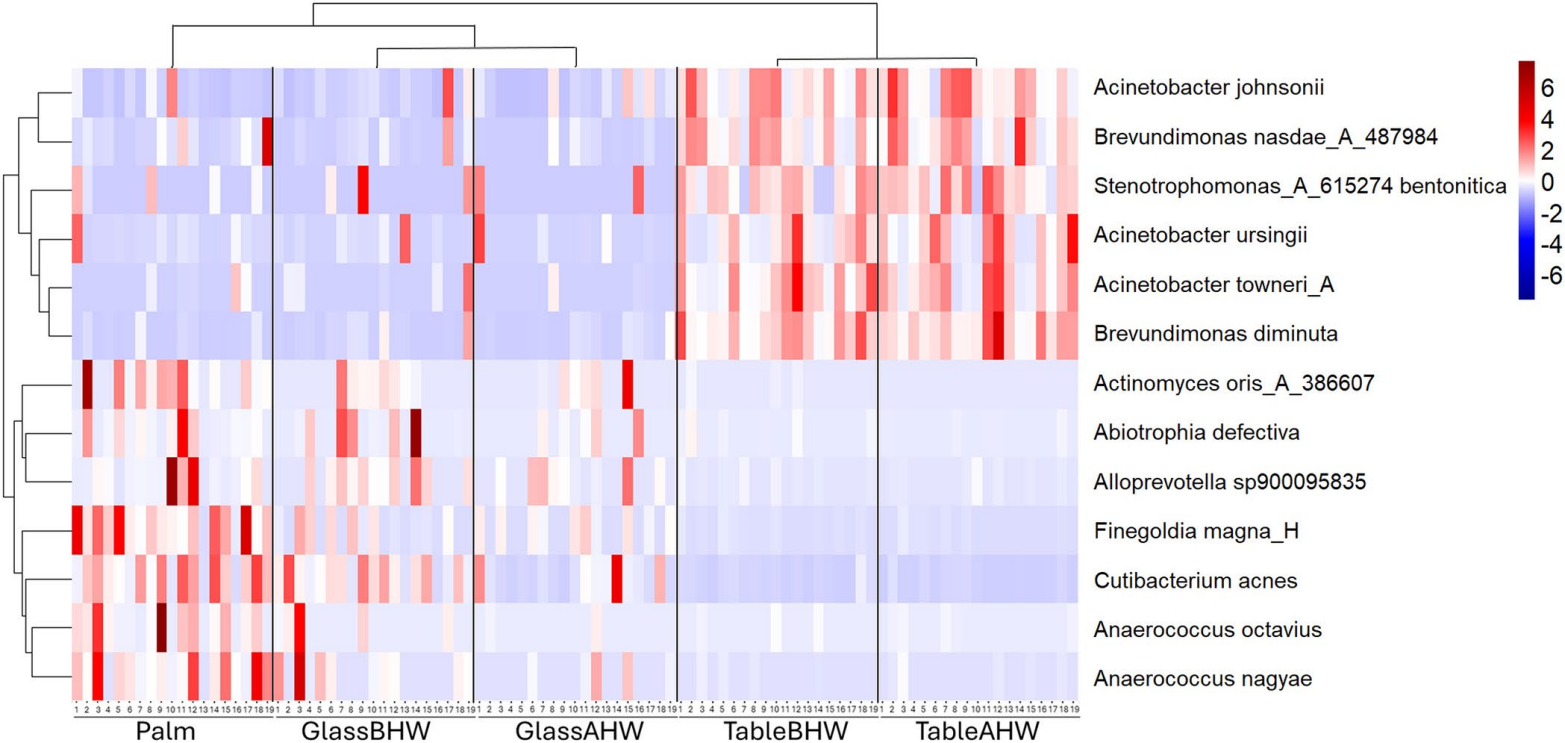
Heatmap of species abundance. This heatmap shows the standardized species abundances across samples clustered based on similar behavior.

Lastly, we focused on bacterial species associated with HAIs or considered as opportunistic pathogens. In Figure 7 we report the heatmap of the species that showed significant differences among experimental groups (full results available on request). In the heatmap, a clear distinction is evident between different bacterial species with contrasting behaviors. In the upper part are grouped the bacterial species showing higher abundance levels in the samples collected from the laminate table in both conditions, with a slight prevalence in the TableAHW group. In contrast, other bacterial species are clustered at the bottom of the heatmap, showing greater predominance on the palm and,in some cases, in samples from glass surface compared to those from the laminate table. In the latter case, a slight decrease in the abundance of bacterial species is observed in the GlassAHW group compared to the GlassBHW. The clusterings of the bacterial species and experimental groups are also displayed within the heatmap, highlighting the abundance relationships between the species and the differences between the different groups of samples. Clustering indicates how bacterial species group together based on their abundance and distribution across experimental groups, providing a visual indication of significant differences observed in the samples across the experimental groups.

**Figure 8:**
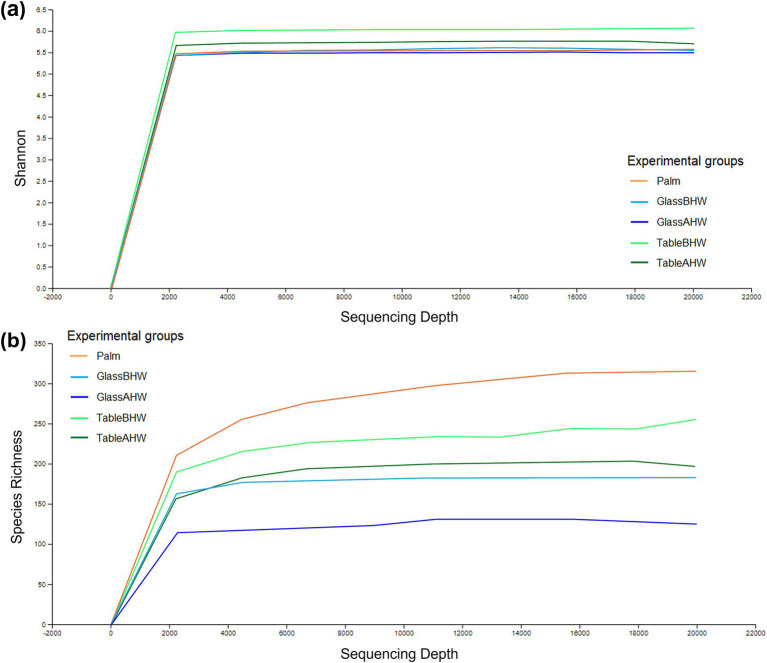
Alpha-rarefaction curves considering a sequencing depth of 20,000. This figure illustrates different curves of alpha rarefaction based on different parameters: **(a)** rarefaction curves based on Shannon index for the different experimental groups and **(b)** rarefaction curves based on species richness for the different experimental groups.

To provide a measure of species richness and their relative distribution within each experimental group, the alpha-rarefaction curves were generated considering a sequencing depth of 20,000 to ensure a comprehensive assessment of microbial diversity. The analysis was conducted on all the 95 samples (19 subjects and 5 experimental groups) and the alpha-rarefaction curves are shown in Figures 8a,b. Shannon indexes (Figure 8a) reached a plateau around 2,000 sequencing depth. The curves obtained from the samples of the TableBHW and TableAHW groups show a higher Shannon index than the other groups. Moving to the alpha-rarefaction curves based on species richness (Figure 8b), it is evident that the palm group shows the highest values, whereas the glass the lowest. Moreover, from both graphs, it is also interesting to note how in the samples taken after hand washing, therefore for the samples of the GlassAHW group compared to those of the GlassBHW group and for the samples of the TableAHW group compared to those of the TableBHW group, there is a reduction in diversity and species richness. This reduction is more evident in the alpha-rarefaction curves based on species richness, which consider only the number of identified species. In contrast, the Shannon index, which also takes into account the abundance of each species, tends to smooth out these differences.

**Figure 9:**
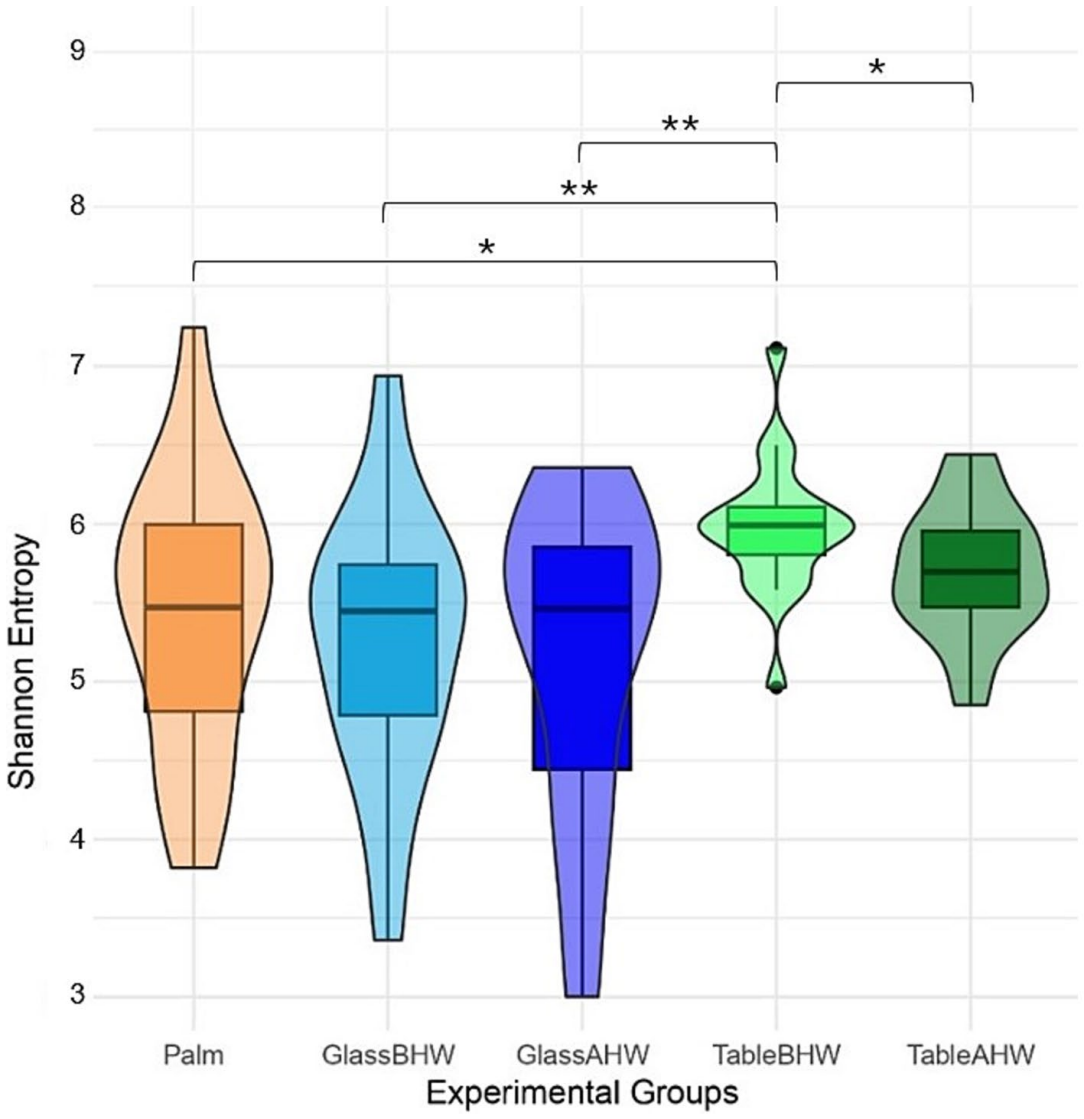
Alpha diversity violine plots. The figure shows five violine plots each one based on Shannon entropy of different experimental groups, whit a sequencing depth of 2,785.

The Shannon index for each sample is reported as violine plots in Figure 9. In this case, to provide a better overview of species richness and equity across all collected samples avoiding data loss, we used a sequencing depth of 2,784 reads. This depth ensures that all samples are preserved, providing a more accurate and complete comparison of microbial diversity across the different experimental groups. In contrast, higher depths could lead to the loss of some samples, thereby compromising the representation of the total microbial variability. The microbial diversity on the dominant palms of the volunteers’ hands showed a nearly symmetric unimodal pattern, with a median around 5.4 and an interquartile range 4.8–6.0. Considering the glass surface, both before and after hand washing, it revealed a median microbial diversity similar to that of the palm. However, the distribution is skewed, with longer lower tail for GlassAHW. Analyzing the laminate table groups, it was observed a slightly higher median microbial diversity but with highly concentrated distributions.

**Figure 10:**
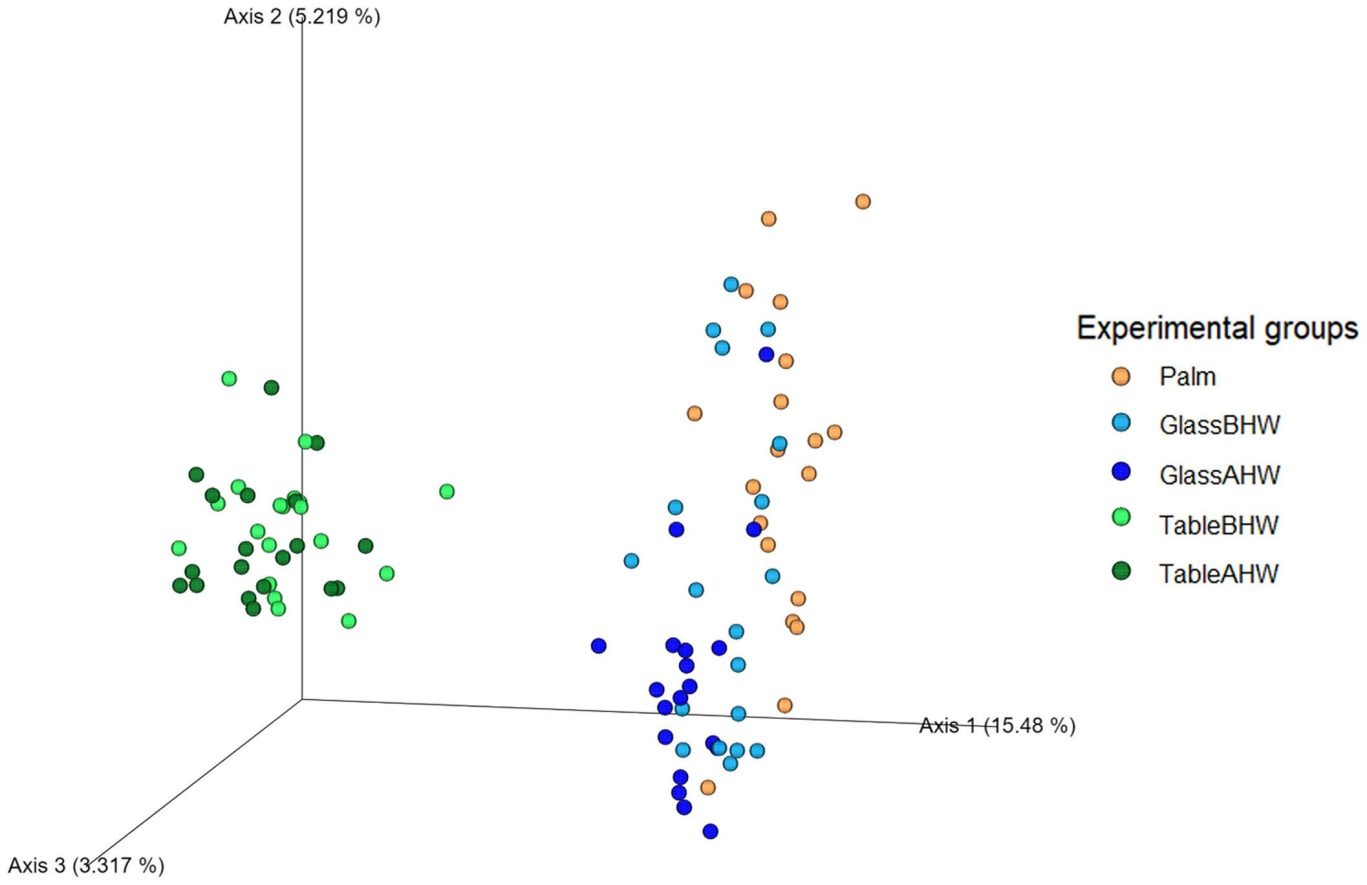
Three-dimensional PCoA plot. The graph represents beta diversity analysis using Aitchison distance where experimental groups are differentiated based on color scale.

The beta diversity analysis through PCoA at the species level is shown in Figure 10. The first axis explains approximately 15.5% of the total variation and shows that samples taken from laminate table surface (TableBHW and TableAHW) had a different microbial composition respect to the samples taken from glass surface (GlassBHW and GlassAHW) and those taken from the hand palm (Palm). The second axis explains approximately 5.2% of the total variation and shows differences in before-after hand washing. The third axis represents a smaller component of the variation (approximately 3.3%), but which still helps to identify slight differences between samples especially among the before and after hand washing conditions particularly for glass samples.

## 4 Discussion

HAIs represent a major problem in global healthcare settings, contributing significantly to patient morbidity and mortality worldwide. Indeed, they are considered a great threat to hospitalized patients and a serious burden on public health (12, 27, 30, 31). Surfaces in hospital environments are recognized as a major reservoir for the transmission of pathogens, facilitating the spread of this type of infection. Understanding how bacteria are transferred from hands to surfaces and evaluating the effectiveness of hand hygiene practices are critical to developing effective prevention strategies (21, 26–28, 38, 59, 60). This study was conducted with the aim of examining the differences in the type and amounts of bacteria transferred from the palm of the dominant hand of different volunteers to two types of surfaces commonly present in hospital environments (glass and laminate table), both before and after hand washing. Through the analysis of the collected samples, we intended to provide a more in-depth understanding of the mechanisms of direct transmission of microorganisms to contribute to developing strategies aimed at preventing HAIs.

First, we evaluated the abundance of Bacteria domain in our five experimental groups (Figure 2). This general overview revealed:

- that the generally observed higher abundance of Bacteria on the palm compared to that on glass and laminate table before hands washing may be explained by the limited transfer and adhesion of Bacteria which depend on the specific physico-chemical characteristics of both the surface and microorganisms (50, 61–63);
- that after hands washing, as expected considering previous results (64–69), the amount of Bacteria deposited on the surface was significantly reduced compared to the situation before hand washing, especially for the glass surface;
- that hand washing does not seem to significantly affect the amount of Bacteria deposited on the laminate table surface, as the quantity of microorganisms found on TableBHW was more similar to those on GlassAHW and TableAHW, rather than to that on GlassBHW. This may be influenced by the type of table surface used for this study: in particular we adopted a laminate table, which is known to be a type of surface with antiseptic features, hence which does not favor attachment and proliferation of microorganisms (49).

Moving to the taxonomic level of the phyla we identified the most abundant taxa were *Actinobacteriota*, *Firmicutes D*, and *Proteobacteria* in Palm (Supplementary Figure 1a). This is in line with previous studies where these phyla were identified as the most abundant phyla present on human hands (70, 71). Considering the human microbiota deposition on the two different surfaces, we observed that the phyla of *Actinobacteriota* and *Firmicutes D*, predominant on the palm, were also predominant on GlassBHW and GlassAHW, whereas the *Proteobacteria* were the most abundant phylum on TableBHW and TableAHW, with levels higher than those of Palm, GlassBHW, and GlassAHW (Supplementary Figures 1a-e; Figure 3). These findings align with previous literature studies, which reported these bacterial phyla as the main components of hand-derived microbiota on different surfaces (50, 72, 73). These results could be explained by the different nature of the phyla and their propensity to better adhere to different surfaces. *Actinobacteriota* and *Firmicutes D* are predominantly Gram-positive bacteria characterized by a thick peptidoglycan cell wall containing teichoic and teichuronic acids, which impart a negative surface charge (74–79). Although glass also tends to have a slightly negative surface charge, due to silanol groups (80, 81), these phyla adhere stably by producing EPS (exopolysaccharides), which facilitate bacterial adhesion, aggregation, and biofilm formation (82). On the other side, *Proteobacteria* are a highly diversified group of Gram-negative bacteria with a thin peptidoglycan layer and an outer membrane containing lipopolysaccharides (LPS), which give a negative charge to the bacteria surface (76, 78, 83, 84). In this phylum, the presence of surface appendages such as flagella and pili along with the production of bacterial cellulose (BC), enhance their ability to adhere to the inhospitable surface of the laminate table (76). Pili mediate initial adhesion on the surface itself, improving colonization capacity, whereas the BC acts as a “molecular glue,” allowing *Proteobacteria* to effectively adhere to surface even with antiseptic features (49, 82, 85–87). These characteristics provide a general explanation for the observed differences in adhesion propensity among different phyla on various surfaces. However, within each phylum, alongside the explained predominant traits, there are also minority species with similar adhesion mechanisms to those found in other phyla. Together, these characteristics contribute to the overall potential adhesion of each phylum, resulting in varying amounts on the different surfaces (76, 87, 88).

Further comparative analyses were conducted at the level of the less abundant phyla. It is interesting to note the greater concordance between the amounts of bacteria for each phylum on the palm and those collected from the glass surface, which showed an opposite trend to those of the laminate table. Also, in this case the differences can essentially be explained by the characteristics of each bacterial phyla (Figure 4). In particular, *Bdellovibrionota E*, the only less abundant phyla that appeared to be predominant on laminate table surface, even if not very present on the palm or on glass, is a Gram-negative bacterium with characteristics similar to *Proteobacteria* (Figure 4a) (89). In our study, we observed that the *Bacteroidota* phylum maintains similar abundance levels among experimental groups. This uniformity in presence, although in low amount compared to the main phyla, suggests that the *Bacteroidota* possess an ability to adapt to various environments, highlighting a notable ecological versatility. This aspect makes them particularly relevant in the context of our research, especially considering their reported involvement in HAIs (Figure 4f) (30, 90).

Since this study aims to prevent HAIs, we decided to analyze the bacteria sampled from volunteer subjects in the various experimental groups, focusing on the main genera known to be associated with HAIs which were detected on our samples (26, 30). The most interesting data to highlight is the preponderance of bacteria belonging to the *Acinetobacter*, *Pseudomonas*, *Staphylococcus*, *Stenotrophomonas*, and *Staphylococcus* genera, with a variable distribution based on the surfaces (Figures 5, 6). In particular, whereas *Acinetobacter*, *Pseudomonas*, and *Stenotrophomonas* are more abundant in samples taken from laminate table, *Staphylococcus* and *Streptococcus* prevail on glass surface, in accordance with what has already been highlighted regarding the specific biological characteristics of these bacteria. Indeed, the first three genera, which prevailed on laminate table, are Gram-negative bacteria, whereas the other two are Gram-positive bacteria (76, 78, 84, 85, 87, 91). These results suggest the need to implement specific hygiene procedures for each type of surface, in order to reduce the presence of these pathogens and, consequently, the impact of the healthcare environment as a reservoir of HAIs-related pathogens (92–95).

Regarding bacterial species associated with HAIs or considered opportunistic pathogens, a similar result was observed (Figure 7). In the upper part of the heatmap, where a greater abundance is represented on the laminate table, Gram-negative bacteria are grouped (91, 96, 97). In the bottom part, instead, Gram-positive bacteria are grouped (98–102). The only exception is *Alloprevotella sp900095635* which, despite being Gram-negative, shows a similar abundance and adhesion to the other Gram-positive bacteria. This is probably due to its intrinsic nature as a host of human microbiota, as in the case for the others reported Gram-positive bacterial species (98, 100–104).

The Gram-positive bacterial species present in the heatmap and *Alloprevotella sp900095635*, despite being common commensals of the human microbiota (98, 100–104), can act as opportunistic pathogens and cause various infections especially in immunocompromised patients. In particular: (i) *Abiotrophia defectiva* can cause bacterial meningitis or infective endocarditis, which may lead to a consequent stroke (99, 103, 105, 106); (ii) *Actinomyces oris* is associated with oral infections and abscesses (98); (iii) *Anaerococcus* spp. may be involved in urogenital tract infections and skin infections (107, 108); (iv) *Cutibacterium acnes* is known to cause acne and may be associated with inflammatory diseases and implant-associated infections (101, 109); (v) *Finegoldia magna* can cause rare infections of bone and joints following orthopedic implant, mechanical prosthetic endocarditis and other skin and soft tissue infections (100, 110, 111); (vi) *Alloprevotella sp900095635* can be associated with oral infection (104, 112). Therefore, the analysis of their persistence in different healthcare surfaces is important to limit their impact on patient health status, especially to protect those who are immunocompromised.In contrast, Gram-negative bacterial species, clustered at the top of the heatmap, are more commonly associated with infections, particularly in healthcare settings. For example, *Acinetobacter* spp. may be isolated in healthcare settings and can cause infections such as pneumonia, catheter-related bloodstream infections, meningitis, and sepsis (96, 113–115); *Brevundimonas* spp., can cause invasive and severe infections of different nature which can involves, among the others, bloodstream and urinary tract (97, 116); and *Stenotrophomonas* spp. is known to cause respiratory and blood infections and to have an high rate of antimicrobial resistance (91, 117, 118). Their correlation with HAIs was already showed at the genus level (Figure 5) (30), except for *Brevundinomas* spp. which is not yet considered among the major HAIs-related pathogens even though it has the ability to cause potentially harmful infection. In this prospective, Ryan et al. suggested to include this bacterial species in healthcare screening and prevention program. However, the overall distinction between Gram-positive and Gram-negative in the heatmap highlights, again, how these bacterial species have different behaviors in adhesion to surfaces. It is crucial to consider these differences when implementing preventive and hygiene measures. Understanding specific adhesion patterns can help to develop more effective strategies for cleaning and disinfection, thereby reducing the risk of HAIs and improving patient safety.

Moreover, it is important to remember that Gram-negative bacteria are considered one of the most significant public health problems due to their resistance to environment and to antibiotics treatments which make them more directly implicated in HAIs compared to Gram-positive bacteria (119, 120). This resistance may explain the reason why, for these bacterial species, a slight increase in abundance is observed on the laminate table after hand washing and, in some samples, also on the glass surface (Figure 7). Although there is a general reduction in the total number of bacteria after hand washing, as we have already described (Figure 2), in this specific case the resistance of Gram-negative bacteria means that they are eliminated less during hand washing than other pathogens. As a result, a slight enrichment of these bacteria occurs on the surfaces of the laminate table and glass after hand washing. In contrast, for other bacterial species, which are generally less resistant, a slight decrease in abundance is observed on both surfaces after hand washing. This can be explained by their less resistant nature, which makes them more susceptible to hygiene procedures. Therefore, a normal hand washing with water and antibacterial soap can reduces the overall number of bacteria on human hands leading to the prevalence of the most resistant bacteria, compared to those which decrease after hand washing, on surfaces. This confirms how much effort still needs to be made to develop effective infection prevention strategies concerning the transfer of bacteria from hands to surfaces within healthcare settings.

The characterization of the microbial communities was performed through the inter-group and intra-group diversity by generating the alpha-rarefaction curves with both Shannon index and species richness in the different experimental groups. Moreover, the Shannon entropy of the samples, across the experimental groups, and the beta-diversity of the samples, with Aitchison distance, were also assessed. The Shannon index takes into account both the richness of the species and their equity, therefore giving an idea of the number of species and their balance from an abundance point of view (121). Since the alpha-rarefaction curves, generated at sequencing depth of 20,000, reach a plateau around 2,000 reads, this analysis ensures a comprehensive assessment of microbial diversity indicating that deeper sequencing does not significantly increase the observed diversity (Figure 8a). It is interesting to note that the average Shannon indexes of the samples taken from the laminate table, both before and after hand washing, are the highest (Figures 8a, 9) despite the species richness in these samples being lower than that of the group of Palm (Figure 8b). However, from Figure 8 is possible to observe how the variability among the individuals is higher if considering palm, GlassBHW, and GlassAHW, whereas the individuals seem to be more similar if considering the groups collected from the laminate table (TableBHW and TableAHW). The laminate table, designed to prevent the adhesion of microorganisms, is characterized by approximately 80% bacteria from the *Proteobacteria* phylum (Supplementary Figures 1d-e). This phylum is known for its species diversity, which may contribute to differentiating the microbial community by increasing species richness for these samples (122). However, this variability, given the alpha-rarefaction curves of species richness for these groups, is not sufficient to explain a higher Shannon index than that of the samples collected from volunteers’ dominant palm. Consequently, this result could be explained by considering the intrinsic nature of the Shannon index, which also considers the uniformity of the species distribution within the TableBHW and TableAHW groups. This result agrees with another study where a high Shannon index was observed in samples with a predominance of the phylum *Proteobacteria* (123). Moreover, it is also important to note that the Palm group has a wide distribution of Shannon index values between samples, indicating great individual variability. Unlike the table groups, for which the variability of the Shannon index between subjects is concentrated around the median value, a result similar to that observed from the palm samples is also observable in the GlassBHW and GlassAHW groups, suggesting a wider individual variability in the microbial component that adheres to this surface (Figure 9). Another relevant aspect (Figures 8, 9) is the decrease in diversity and species richness of the samples collected from the glass and the table after hand washing compared to those of the samples collected from the same surfaces before hand washing, suggesting a role of hand washing in reducing microbial diversity.

The beta diversity analysis using the Aitchison distance supports previous results obtained from the alpha diversity analysis. The clear separation of groups in the PCoA plot (Figure 10) confirms significant differences in microbial composition between the analyzed samples. This is particularly evident in the samples taken from the laminate table compared to the other groups, suggesting, as already highlighted, that the differences in microbial engraftment on the laminate table are closely related both to the characteristics of the surfaces and the adhesion capabilities of the different bacteria. Further confirmation of the impact of hand washing on microbial diversity is observed when considering axis 2 of the PCoA plot, where samples from the GlassAHW and TableAHW groups are more concentrated toward the bottom compared to those from the GlassBHW and TableBHW groups, which show a wider distribution along this axis toward the top.

Despite the significant findings, we are aware of the inherent limitations of our study that need to be considered. First, the types of surfaces and the use of a small number of healthy volunteers may not be representative of different healthcare environment, and it is therefore difficult to generalize the results. Furthermore, the analyses were conducted considering only time zero and do not take into account the dynamics that the deposited microbial community may have over time. Therefore, although this study provides a basis for understanding the mechanisms of direct transfer on different surfaces in the healthcare context, there is a clear need to conduct future studies with a larger and more diverse sampling, both in terms of individuals and surfaces, for a more detailed healthcare microbiota characterization. Additionally, it would be useful to conduct further research considering different timescales to confirm the persistence of the highlighted bacteria and evaluate the effectiveness of different hygiene procedures, both for hand washing and surface disinfection, in order to develop more targeted and effective interventions. Moreover, the consideration of also viral and fungal contamination could be evaluated, since they are also relevant to HAIs. However, this work is intended to represent a starting point for future investigations and improvements in infection control practices.

## 5 Conclusion

The study revealed the highest bacterial abundance in the Palm group compared to the other groups with a significant reduction after hand washing, especially in GlassAHW group. The analysis of microbial adhesion on different surfaces before and after hand washing revealed the presence of HAIs-related bacteria, with Gram-positive characterizing Palm and Glass groups and Gram-negative characterizing Table groups. Although the specific bacterial species identified in our microbial samples not always correspond to the most common pathogen bacteria associated with HAIs, their environmental persistence still suggests a potential risk of environmental contamination and pathogen transmission within the healthcare environment, given their potentiality to cause severe infection in humans. Our results highlight the importance of microbial surveillance and preventive measures, not only for well-known bacterial species, but also for other opportunistic bacteria with potentially pathogenic characteristics, to ensure safe healthcare environments and reduce the risk of HAIs. It is important to focus on achieving improved hygiene practices and targeted interventions to reduce the presence of bacteria on healthcare surfaces. Therefore, further studies on hand to surface microbial contamination are essential in order to implement more effective guidelines for surface disinfection and hand washing and to promote the safety of patients and healthcare professionals through the adoption of more informed and targeted preventive measures.

## Data Availability

The original contributions presented in the study are included in the article/Supplementary material, further inquiries can be directed to the corresponding author.
