# Supplementary material for: Hand-to-surface bacterial transfer and healthcare-associated infections prevention: a pilot study on skin microbiome in a molecular biology laboratory
Source: Front Med (Lausanne). 2025 Mar 21;12:1546298. doi: 10.3389/fmed.2025.1546298 (PMC11970135; doi:10.3389/fmed.2025.1546298)
Supplement: Supplementary file 2 [file Data_Sheet_1.pdf]

Supplementary Material

1.1 Supplementary Figures

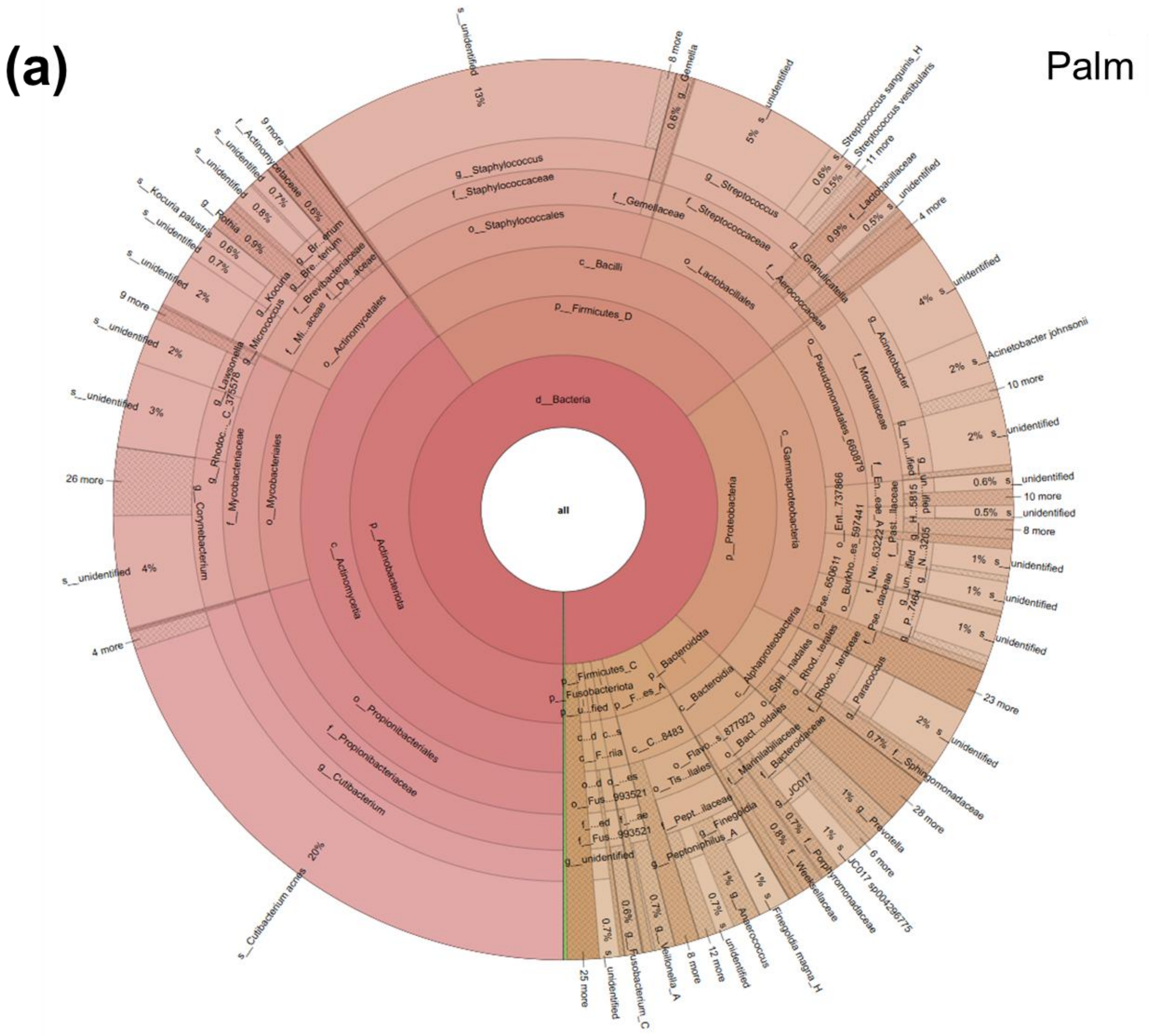

Supplementary Figure 1. Krona chart of bacteria recovered in the different experimental conditions from 19 volunteers. (**a**) Krona chart representing the average relative bacterial composition across 19 volunteers from samples collected from the individuals' dominant palms.

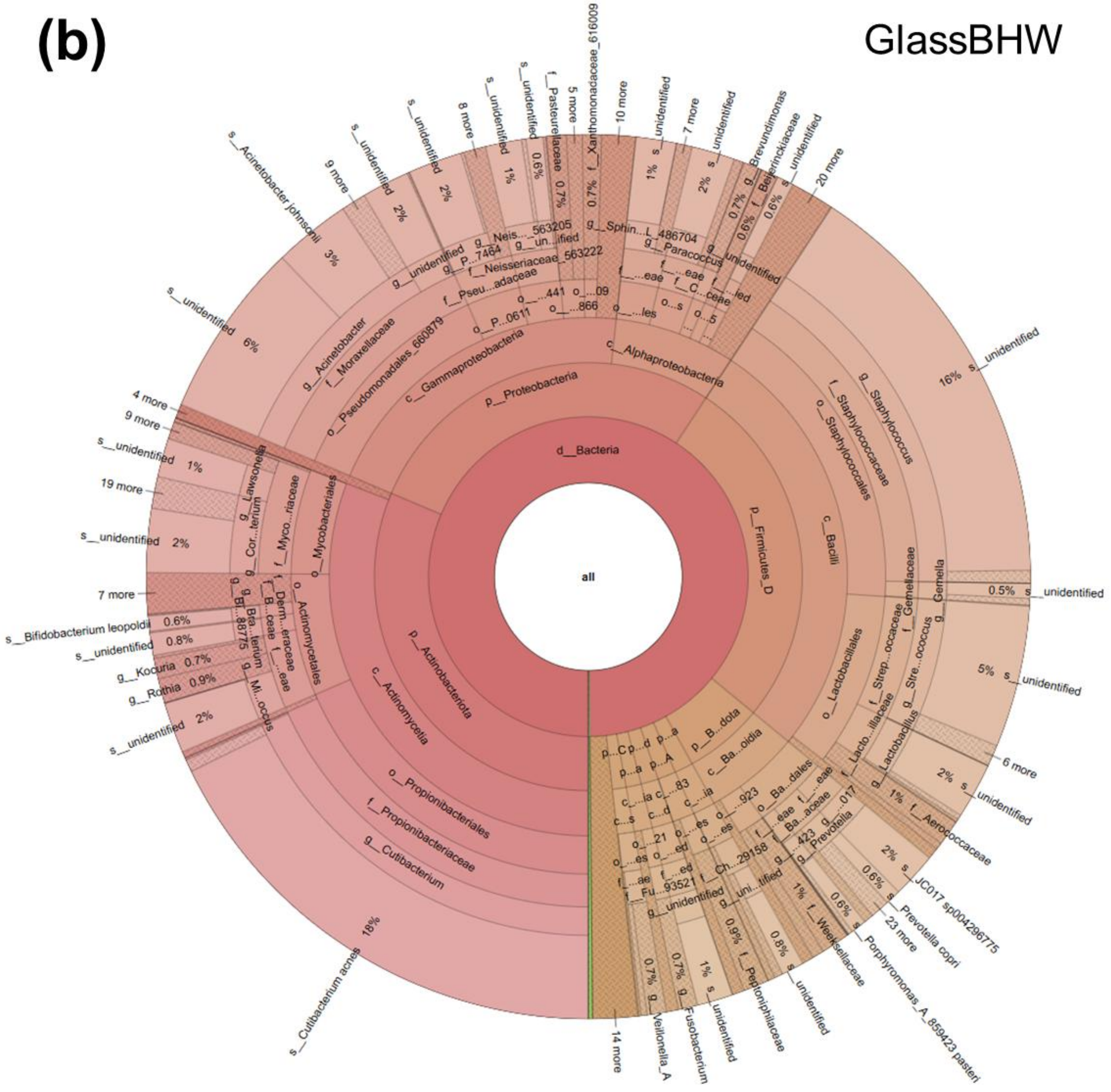

Supplementary Figure 1. (**b**) Krona chart representing the average relative bacterial composition across 19 volunteers from samples collected from the glass after deposition of dominant palms microbiota before hand washing.

(c)

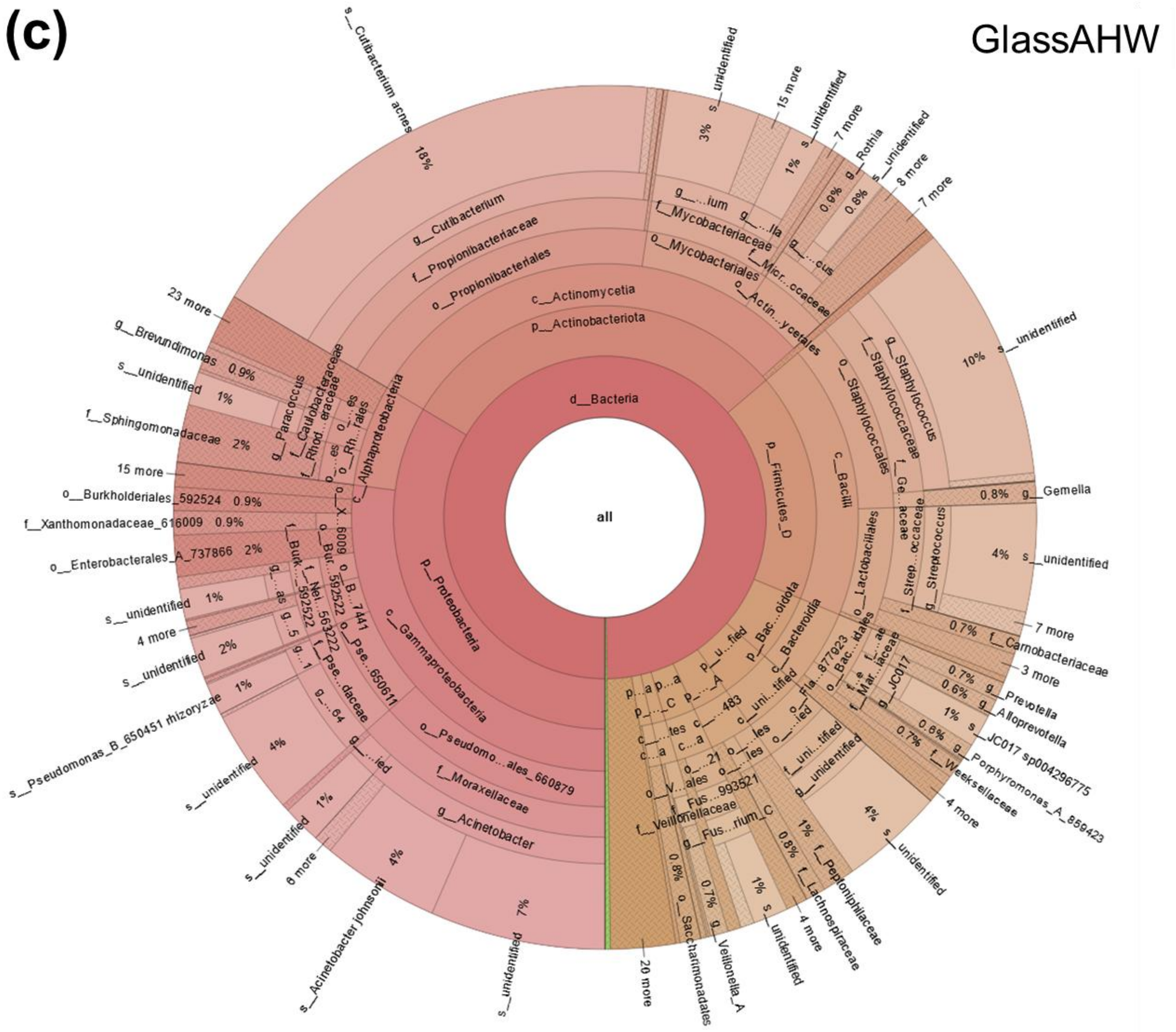

Supplementary Figure 1. (**c**) Krona chart representing the average relative bacterial composition across 19 volunteers from samples collected from the glass after deposition of dominant palms microbiota and hand washing.

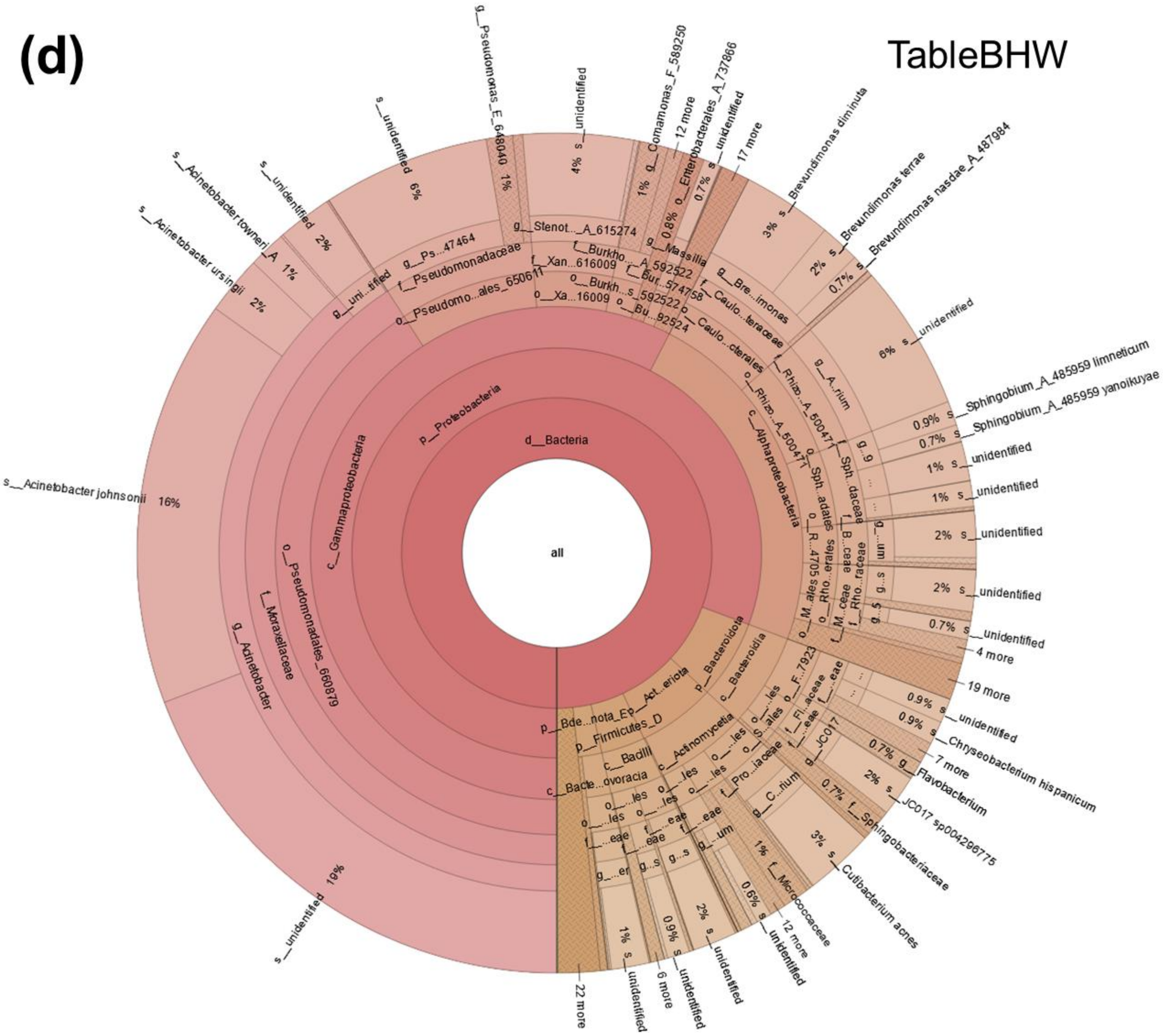

Supplementary Figure 1. (**d**) Krona chart representing the average relative bacterial composition across 19 volunteers from samples collected from the laminate table after deposition of dominant palms microbiota before hand washing.

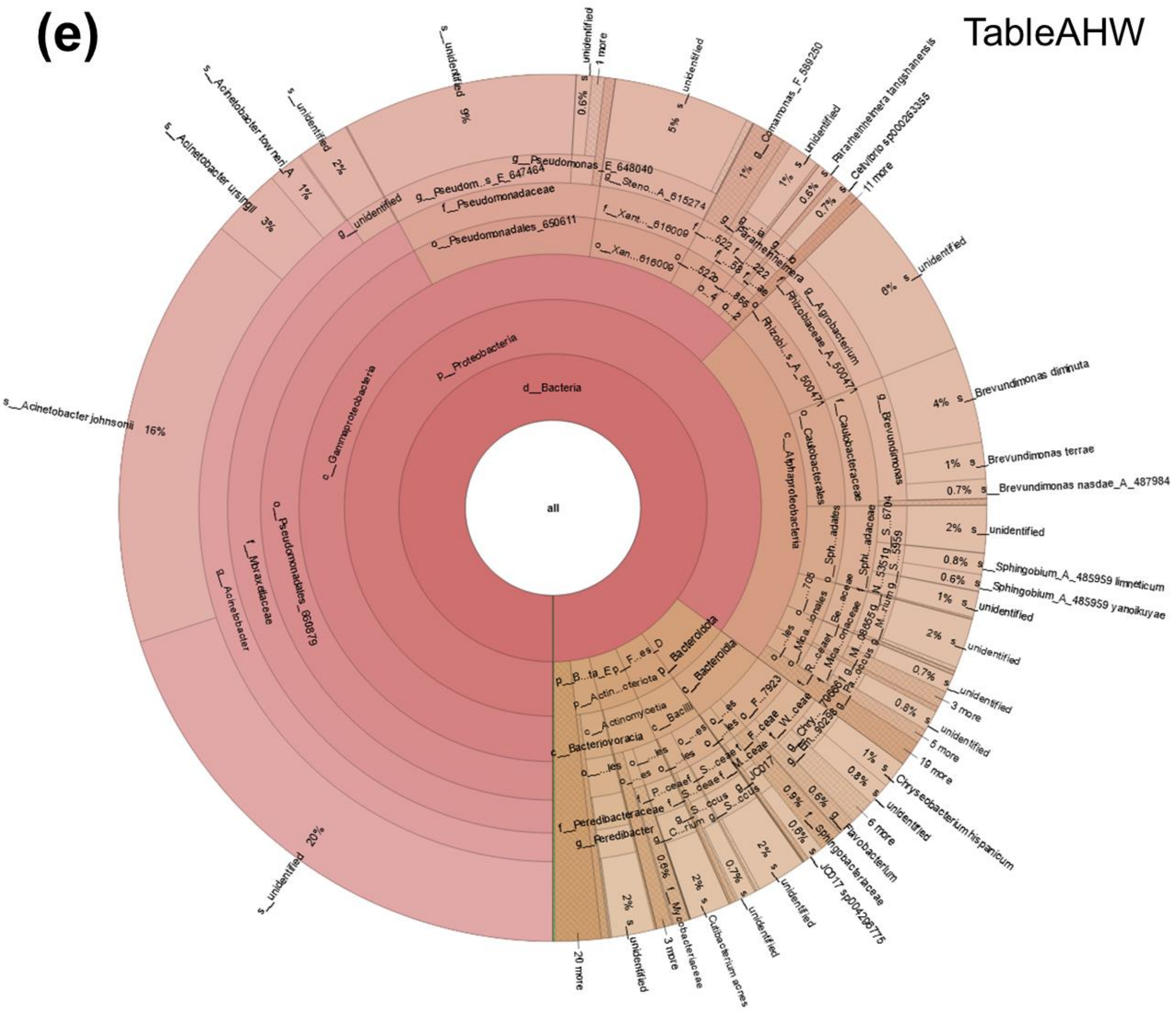

Supplementary Figure 1. (**e**) Krona chart representing the average relative bacterial composition across 19 volunteers from samples collected from the laminate table after deposition of dominant palms microbiota and hand washing.
